# Animal Research beyond the Laboratory: Report from a Workshop on Places Other than Licensed Establishments (POLEs) in the UK

**DOI:** 10.3390/ani10101868

**Published:** 2020-10-13

**Authors:** Alexandra Palmer, Beth Greenhough, Pru Hobson-West, Reuben Message, James N. Aegerter, Zoe Belshaw, Ngaire Dennison, Roger Dickey, Julie Lane, Jamie Lorimer, Kate Millar, Chris Newman, Kirsten Pullen, S. James Reynolds, Dominic J. Wells, Matthew J. Witt, Sarah Wolfensohn

**Affiliations:** 1 School of Geography and the Environment, University of Oxford, Oxford OX1 3QY, UK; beth.greenhough@ouce.ox.ac.uk (B.G.); reuben.message@ouce.ox.ac.uk (R.M.); jamie.lorimer@ouce.ox.ac.uk (J.L.); 2 School of Sociology and Social Policy, University of Nottingham, Nottingham NG7 2RD, UK; Pru.Hobson-west@nottingham.ac.uk; 3 National Wildlife Management Centre, Animal and Plant Health Agency, Sand Hutton, York YO41 1LZ, UK; James.Aegerter@apha.gov.uk (J.N.A.); Julie.Lane@apha.gov.uk (J.L.); 4 PDSA Nottingham, Dunkirk Road, Nottingham NG7 2PH, UK; zoevet@hotmail.com; 5 Biological Services, University of Dundee, Dundee DD1 5EH, UK; n.dennison@dundee.ac.uk; 6 Army Ornithological Society (AOS), Aldershot GU11 1PS, UK; roger.dickey52@gmail.com (R.D.); J.Reynolds.2@bham.ac.uk (S.J.R.); 7 Centre for Applied Bioethics, School of Biosciences and School of Veterinary Medicine and Science (SVMS), University of Nottingham, Nottingham LE12 5PF, UK; kate.millar@nottingham.ac.uk; 8 Wildlife Conservation Research Unit, The Recanati-Kaplan Centre, Department of Zoology, University of Oxford, Oxford OX13 5QL, UK; chris.newman@zoo.ox.ac.uk; 9 Wild Planet Trust, Paignton Zoo, Totnes Road, Paignton TQ4 7EU, UK; kirsten.pullen@wildplanettrust.org.uk; 10 School of Biosciences, University of Birmingham, Birmingham B15 2TT, UK; 11 Department of Comparative Biomedical Sciences, Royal Veterinary College, London NW1 0TU, UK; dwells@rvc.ac.uk; 12 College of Life and Environmental Sciences, University of Exeter, Exeter EX4 4QD, UK; M.J.Witt@exeter.ac.uk; 13 School of Veterinary Medicine, University of Surrey, Guildford GU2 7AL, UK; s.wolfensohn@surrey.ac.uk

**Keywords:** animal research, animal welfare, farms, fisheries, governance, policy, veterinary medicine, wildlife, zoos

## Abstract

**Simple Summary:**

Animal research conducted outside of the laboratory faces various unique challenges, but has received only limited attention in terms of official guidelines, support, and statistics. To improve understanding, we held a workshop bringing together experts familiar with a variety of nonlaboratory animal research contexts (e.g., wildlife field sites, farms, fisheries, veterinary clinics, zoos). We collectively identified five key areas that we propose require further discussion and attention, which we present in this paper. While the workshop focused on research in the UK, our conclusions may have implications for similar work overseas.

**Abstract:**

Research involving animals that occurs outside the laboratory raises an array of unique challenges. With regard to UK legislation, however, it receives only limited attention in terms of official guidelines, support, and statistics, which are unsurprisingly orientated towards the laboratory environment in which the majority of animal research takes place. In September 2019, four social scientists from the Animal Research Nexus program gathered together a group of 13 experts to discuss nonlaboratory research under the Animals (Scientific Procedures) Act (A(SP)A) of 1986 (mirroring European Union (EU) Directive 2010/63/EU), which is the primary mechanism for regulating animal research in the UK. Such nonlaboratory research under the A(SP)A often occurs at Places Other than Licensed Establishments (POLEs). The primary objective of the workshop was to assemble a diverse group with experience across a variety of POLEs (e.g., wildlife field sites, farms, fisheries, veterinary clinics, zoos) to explore the practical, ethical, and regulatory challenges of conducting research at POLEs. While consensus was not sought, nor reached on every point of discussion, we collectively identified five key areas that we propose require further discussion and attention. These relate to: (1) support and training; (2) ethical review; (3) cultures of care, particularly in nonregulated research outside of the laboratory; (4) the setting of boundaries; and (5) statistics and transparency. The workshop generated robust discussion and thereby highlighted the value of focusing on the unique challenges posed by POLEs, and the need for further opportunities for exchanging experiences and sharing best practice relating to research projects outside of the laboratory in the UK and elsewhere.

## 1. Introduction

The Animals (Scientific Procedures) Act (A(SP)A) of 1986 is the primary mechanism for regulating invasive scientific research in the UK, with any “protected” animal, i.e., vertebrates and cephalopods when either the species-specific gestation or incubation period has elapsed (in the cases of mammals, birds, and reptiles), or when they become capable of independent feeding [1]. It is the UK’s approximate equivalent legislation to EU Directive 2010/63/EU. While the majority of research licensed under the A(SP)A is conducted in laboratories, the A(SP)A extends to research conducted outside of the laboratory, such as at practicing farms—i.e., farms used for agricultural and fish production, rather than those used exclusively for research—veterinary clinics, zoos, and wildlife and fisheries field sites, which might be built structures (e.g., field stations, enclosures designed to keep animals semicaptive) or open-air locations (e.g., in woodlands, on boats). Such venues outside of the laboratory are often classified under the A(SP)A as Places Other than Licensed Establishments (POLEs). Licensed user establishments require the presence of specific personnel and systems of control, and provision of particular environmental conditions. Although some nonlaboratory settings can meet these requirements and become licensed user establishments (e.g., farms and animal hospitals associated with universities), POLEs are locations where A(SP)A-regulated research occurs where all of these regulations cannot be met. Research at POLEs is explicitly the exception rather than the rule, since researchers must justify why they are unable to conduct the research within a licensed user establishment [1,2].

There is increasing recognition that POLEs and comparable nonlaboratory work overseas—i.e., work that would be classified as A(SP)A-regulated research at a POLE if it occurred in the UK—present their own unique practical and ethical challenges compared with laboratory-based research. In many countries, nonlaboratory research can face less designated supervision than research undertaken within institutional laboratories, as specialized staff such as veterinarians and animal caregivers are not always present, and sites may be remote and difficult for regulators to inspect [3]. Wildlife and fisheries researchers in the USA have highlighted how their work involves a large and confusing array of permits, in addition to those secured under the primary laws regulating animal research (the Animal Welfare Act of 1966 and the Animal Welfare Regulations, enforced by the Animal Care division of the Animal and Plant Health Inspection Service (APHIS) of the United States Department of Agriculture (USDA)) [4]. Furthermore, wildlife and fisheries researchers have highlighted that their work may require ethical considerations beyond individual animal welfare given that research in the wild may also affect conspecifics, other species, and broader ecosystems [5,6,7,8]. It has also been proposed that aspects of the 3Rs (the commitment to *reduce* the number of animals used, *refine* methods to minimize harm, and *replace* animal research with alternatives whenever possible) may be more challenging in wildlife studies around the world. For example, it can be challenging to capture and sample sufficient wild animals to make the study viable, and postprocedure welfare monitoring is difficult when working on free-living animals that are subsequently released [3,9].

Research at POLEs also raises questions about which activities fall under the A(SP)A and similar regulations, since the procedures undertaken are often only marginally invasive and fall near the “lower threshold”, which must be met or exceeded for a project to be regulated under the A(SP)A. This lower threshold is defined as causing “pain, suffering, distress or lasting harm equivalent to, or higher than, that caused by inserting a hypodermic needle according to good veterinary practice” [1]. EU Directive 2010/63/EU “on the protection of animals used for scientific purposes” took inspiration from the A(SP)A, and was transposed into UK law in 2013. For this reason, a similar threshold applies when determining the regulation of animal research across the EU, although local implementation may vary (L 276/39, Article 3 [10]). Yet, it can be difficult to judge when procedures meet this threshold. For example, when assessing the welfare effects of biotelemetry devices on birds it is advisable to consider the behavior and ecology of the focal species, the attachment method and device location, and tag weight and shape [11,12,13,14], making it potentially difficult to assess when proposed tagging of birds falls under the A(SP)A [15,16]. Furthermore, in wildlife studies it may be the method of restraint or sedation rather than the attachment of a device that exceeds the lower threshold [2]. As research projects at POLEs often sit close to this lower threshold, examining the regulation of research with nonlaboratory animals also raises questions about the regulation (or lack thereof) of animal work apparently outside of the A(SP)A, such as that often conducted by citizen scientists and ecological consultants [15,17,18]. Animal work at POLEs can also often include, or closely resemble, nonscientific activities such as animal husbandry and veterinary treatment, which are explicitly excluded from the remit of the A(SP)A [1]. Distinguishing research from husbandry and veterinary treatment may be challenging. Discussions within the veterinary community have therefore focused on the distinction between Recognised Veterinary Practice (RVP) and research [19,20,21,22,23,24,25,26]. Similar issues may arise overseas, with researchers in Sweden, for example, pointing to the difficulty of determining when nonlaboratory research (e.g., with wildlife) is undertaken for research or management purposes, a decision which has important implications for regulation and oversight [17].

Discussions around nonlaboratory research may also focus on unique ethical challenges posed by this work. For example, discussions within the veterinary community concern the prevalence [27] and ethics of veterinary clinical trials and novel veterinary treatments with companion animals [28,29,30,31,32,33]. Some ethical questions relate to relationships between veterinary researchers and the owners of animals, such as how much information owners are offered about what enrolling their pet in a clinical trial involves and their alternative options [30], and how decisions about experimental treatments that may extend an animal’s life versus euthanasia are reached [29]. This last example highlights how the unique challenges of research at POLEs may arise from the presence of animal owners and other members of the public, whose permission may be required for research to proceed. This reflects the point regularly made in social studies of science, that while laboratories tend to be contained and controlled spaces, usually only accessible to those involved in research and research support, nonlaboratory spaces of research, or “fields”, are often multiuse spaces where various members of the public and other stakeholders are colocated [34,35,36].

Despite these ongoing discussions about the unique challenges and questions arising in nonlaboratory research, POLEs remain largely ignored in wider discussions about UK animal research. For example, there is no published information on the types and distribution of research venues that might be defined as a POLE, the number of research projects carried out at POLEs, and the number of animals studied specifically at POLEs in the UK. Figures on the number of non-A(SP)A projects and animals studied in research outside of the laboratory, such as subthreshold studies involving trapping and marking of wildlife, are also largely unknown. Furthermore, while the laboratory animal arena features numerous specialized networks intended to share information and provide support for those involved (e.g., in the UK, the Laboratory Animal Science Association (LASA), Laboratory Animals Veterinary Association (LAVA), and Institute of Animal Technology (IAT)), fewer such networks exist for nonlaboratory research at POLEs. Some commentators have also observed that most guidelines apply more appropriately to the laboratory rather than to companion animals [28] and wildlife [9], although this may be changing, as heralded, for example, by introduction in 2016 of guidelines under the A(SP)A targeted at researchers working with animals taken from the wild [2]. Similar issues may arise overseas, with researchers in the USA, for example, describing a perceived lack of attention from Institutional Animal Care and Use Committees to the unique ethics and practical challenges encountered in nonlaboratory research [4,5,8,37,38].

Research for the Animal Research Nexus project (AnNex; https://animalresearchnexus.org/) uses methods from social sciences, humanities, and public and stakeholder engagement to understand emerging issues in animal research and helps to encourage communication across the sector [39]. One substrand of AnNex focuses on animal research at POLEs; this research has suggested that many who work at POLEs would appreciate further discussion about the unique challenges that POLEs present to the researcher compared with laboratory settings. For this reason, a workshop was convened to explore the practical, ethical, and regulatory challenges encountered by researchers working at POLEs. The goal of the workshop was to bring together a diverse array of people with expertise from a variety of different POLEs to identify key challenges faced in their work, and discuss areas that may require attention from policy-makers and researchers. The aim of this paper is to describe key themes that emerged from the workshop discussions and offer recommendations for addressing outstanding questions and issues relating to animal research conducted at POLEs.

## 2. Materials and Methods

The workshop organizers (AP, BG, PHW, RM)—all social scientists involved in research for the AnNex project—recruited contributors via email on the basis of their expertise in a specific area relating to research at POLEs, as determined by a review of literature and the organizers’ professional networks (see Table S1, for further detail on organizers’ and contributors’ expertise and roles in the workshop). Not all experts contacted were able to attend the workshop in person. The aim was to recruit contributors who could collectively identify and analyze key issues in several types of POLEs—namely farms, fisheries, zoos, veterinary clinics, and wildlife field sites—and possessed a diverse range of backgrounds and areas of expertise, including regulators, veterinarians, researchers, ethicists, social scientists, and citizen scientists. However, it should be noted that because no information is available on the kinds of sites that are classified as POLEs, it is possible that the workshop neglected some areas. 

The workshop was invite-only, and purposively designed to be small in order to maximize the sharing of experiences. Attendees spoke from their own disciplinary backgrounds and experiences, but did not necessarily speak on behalf of their organizations. The workshop took place at Keble College at the University of Oxford on the 30th of September and 1st of October, 2019. All invited speakers gave verbal presentations in person, except CN who spoke remotely via a prerecorded presentation. J. Lane gave a public plenary on wildlife research ethics and regulation at the start of the meeting. The workshop the following day was organized as four panel discussions featuring 2–3 presentations followed by questions and discussion (see Table S1): (1) introduction and wildlife (ND, SJR/RD, JA); (2) marine research, zoos, and farms (MW, KP, KM); (3) veterinary clinics (DW, ZB); and (4) reflections (SW, J. Lorimer). While presentations in panel 4 (reflections) were 10 min each, others were each 20 min and were requested to respond to the following questions, which were prepared based on emerging themes from the AnNex research on POLEs, and on the broader aims of the AnNex program. These questions (as outlined in [40]) were:

- How does the category of animal (e.g., pets, wild animals, those housed in zoos or farms) shape ethical obligations, veterinary treatment, and humane end-points? How does the A(SP)A manage these ethical obligations and influence decisions?
- How are boundaries drawn between work under and outside of the A(SP)A, and how do these boundaries shape research and animal welfare practice and regulation?
- How do the general public and other stakeholders engage with research at POLEs?
- How does taking scientific research with animals out of the laboratory shape the knowledge produced?
- How is research with animals outside of the laboratory best regulated?

Throughout the workshop and as preagreed, all attending members of AnNex responsible for organizing the workshop (AP, BG, PHW, RM) took notes. All attendees at the workshop agreed to coproduce this synthesized work as coauthors. The workshop organizers, with input from all attending, identified key themes based on consolidated notes and presentation slides shared by speakers. Descriptions of key themes were compiled in the form of summary notes, which were approved and modified by speakers prior to sharing online via the AnNex website [40]. In the following sections we expand on these key themes, drawing on discussions during the workshop and wider reading, and offer recommendations for addressing outstanding questions and issues relating to research carried out at POLEs.

## 3. Results and Discussion

We begin by describing six key themes that emerged from the workshop, relating to: (1) practical and ethical contrasts between research at POLEs and in the laboratory; (2) transparency and profile of POLEs; (3) ethical review; (4) training, competency, and support; (5) regulation and cultures of care; and 6) defining science. Five key areas emerged from these themes that we propose require further discussion and attention, which we describe in Section 4 (Conclusions). 

### 3.1. Practical and Ethical Contrasts between Research at POLEs and in the Laboratory

Discussion focused on practical limitations encountered at POLEs that are not present in the laboratory. Rather than being purpose-bred laboratory animals, typically animals studied in research at POLEs are either free-living or client-owned and tend to only be research animals regulated by the A(SP)A for a limited period, i.e., while research is ongoing. This means that animals may be brought under legal control of the Act in variable health and body condition, such as when trapped and then subsequently released to the wild following procedure(s). However, offering veterinary treatment to free-living animals in low body condition (e.g., suffering from a naturally occurring disease) may interfere with the aims of the study and, therefore, according to the predefined research protocols, treatment would be minimized. Logistical challenges to providing high-quality veterinary treatment may arise due to limited resources, such as restricted access to veterinary equipment, and environmental challenges, such as remote locations and variable weather conditions [3,9]. Furthermore, it was observed that attending to the 3Rs can be difficult in studies of free-ranging animals [9], since wide genetic variation might necessitate large sample sizes, but it can simultaneously be difficult to catch and track enough animals to ensure that data are scientifically meaningful (posing challenges to *reduction*). Furthermore, *replacement* is not possible where studies focus on animal behavior and ecology under natural conditions.

**Figure 1:**
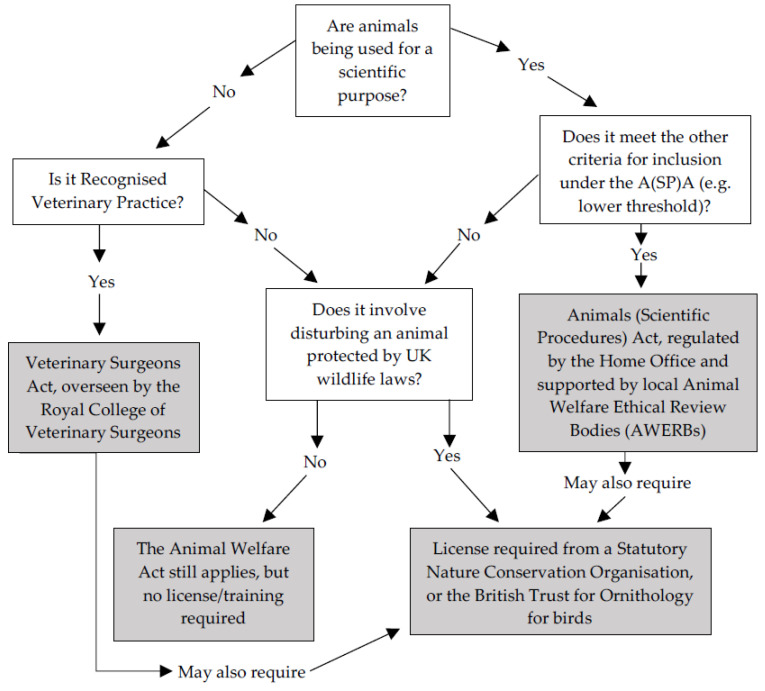
Decision tree, created by the authors, showing how to determine if work with animals in nonlaboratory settings falls under the A(SP)A or other UK laws. Information about laws is highlighted in grey boxes; arrows show conclusions or follow-on questions based on answers.

The challenges posed by working under a wide variety of regulations were also discussed. To give an example, researchers working with free-living birds in the UK under the A(SP)A will often require licenses from the Home Office, the British Trust for Ornithology (BTO), and a Statutory Nature Conservation Organisation (SNCO: Natural England, Scottish Natural Heritage, and Natural Resources Wales). Additional permits may also be required if, for example, researchers focus on wildlife disease or on non-native invasive species, which may require additional licensing from the Animal and Plant Health Agency (APHA) (see [31] for discussion of similar challenges in the USA). Where none of these permits applies, the Animal Welfare Act (2006) still does, which focuses on both preventing animal cruelty and ensuring that animal welfare needs are met and applies when animals are “under the control of man” (including when they are caught in a trap) [9]. Figure 1 illustrates how work with animals in farms, fisheries, veterinary clinics, zoos, and the wild is deemed to fall under the A(SP)A or other relevant laws.

While contributors agreed that practical implementation of the A(SP)A can be dramatically different depending on the setting, there was less agreement on whether the ethical issues raised in and outside of the laboratory differ or remain consistent. On the one hand, some argued that in all settings ethical issues are best resolved via a fundamental harm−benefit calculus, the principles of which remain constant and are embedded in the A(SP)A [41]. On the other hand, it was proposed that research in the wild, in the veterinary clinic, and on the farm can involve a wide range of additional ethical considerations relating to animal welfare, scientific output, and the presence of additional social actors. In the latter case for example, the consent of animal owners is required for research conducted under the A(SP)A in veterinary clinics and on farms, the challenges of which were discussed in the workshop. Furthermore, there may be periods (e.g., between those of data collection or experimental treatments) when animals legally remain under the A(SP)A, and therefore are entitled to care such as daily health checks as specified in the Act, but have been released back into the wild or are under the care of their owners rather than research staff. In the latter case, researchers are required to think about how best to explain their work to clients (e.g., pet owners, farmers), and how to respond if the preferences and behavior of the animal owners do not align with those of the researchers.

Similar questions may arise in wildlife research if members of the public encounter an active research site, or a marked animal. As raised by workshop attendees, this may prompt security concerns, particularly if public assumptions about the nature of the research can potentially be negative. Even where public responses to the research are positive, their presence may pose other risks such as to biosecurity and research disruption. Furthermore, research animals may be killed accidentally (e.g., by cars), or by third parties, for example with wildlife species that are hunted or subject to population controls, which may compromise study continuity and sample sizes. Workshop discussions also dwelled on the point that wildlife researchers must think beyond the welfare of the individual animal to consider the impacts of their research on wider populations and ecosystems. For example, nontarget animals may be caught in traps, and it may be desirable (where possible) to avoid conducting research on animals with dependent young [9].

### 3.2. Transparency and Profile of POLEs

The workshop discussions also focused on the claim that the public can conflate “animal research” with work in laboratories. This idea has been presented elsewhere, such as by members of the zoo community concerned that this conflation will lead to suspicion of zoo research [42]. Workshop attendees argued that this view, if widely held by publics, would represent a double-edged sword for animal research conducted at POLEs. On the one hand, it may mean that public perceptions of both wildlife and clinic-based veterinary research tend to be more positive than of laboratory-based biomedical research (but more empirical work on this is needed). This could reflect a reality that nonlaboratory research often involves minimally invasive activities; in wildlife studies for instance, the most invasive activity undertaken in the course of research may be trapping, sedation, or collection of a blood sample, which might be perceived as minimally invasive by the general public (although more data on the level of harm in POLE- versus laboratory-based research are needed). Furthermore, free-living study animals are usually released after research procedures, which may be perceived as more ethically desirable than studies in which research animals are humanely killed at the end of the research protocol. It was also pointed out in the workshop that in veterinary clinical research the net result for animal welfare should be positive, since clinical research involves treating animals already suffering from a naturally occurring condition, rather than creating animal models with a condition and then attempting to treat them. Veterinary clinical research and conservation-focused wildlife research might also benefit the study species, in contrast to biomedical research for example when animals are more typically used for the benefit of humans. For this and other reasons, veterinary clinical research is often represented publicly in a positive light [43,44,45,46]. That said, neither wildlife nor veterinary clinical research is free from risks to animal welfare [3,9,28,29,30]. 

On the other hand, the conflation of “animal research” with research in laboratories may contribute to a lack of attention to research conducted on animals at POLEs. Discussions in the workshop dwelled on examples of the scarcity of published statistics, guidance documents, and support networks for animal researchers who conduct work at POLEs. To give an example in relation to wildlife research, based on an analysis of the species for which A(SP)A licenses were issued between 2013 and 2016, it was estimated that requests were issued to undertake research on approximately 500,000 wild animals [C. Soulsbury, University of Lincoln, personal communication, 18 March 2020]. However, this is an estimate rather than a verifiable total, and it is unclear how many of these licenses were issued for work at POLEs—i.e., catch, use, and release of animals in the wild—compared with cases when free-living animals are caught, kept and used in a laboratory, and then either humanely killed or released back to the wild. However, we expect wildlife research at POLEs to be more common than wildlife research in laboratories, as restrictions on the latter (e.g., around quarantine when taking free-ranging animals into pathogen-free laboratories, and conditions for release back to the wild) may make such research challenging [2,47]. Furthermore, it remains unclear how many licenses were issued for research on companion, farm, fishery, and zoo animals at POLEs. Statistics are similarly incomplete for subthreshold—i.e., non-A(SP)A—research with wildlife. While the BTO has reported that 982,858 birds were ringed by licensed ringers in 2018 [48], SNCOs responsible for issuing permits under the Wildlife and Countryside Act (WCA) collect but do not publish statistics on the number of animals processed under these licenses, which are issued for any disturbance (including for research) of animals protected under the WCA. However, more thorough statistics are collected for certain species, such as European badgers (*Meles meles*) under the Protection of Badgers Act (1992).

Based on this lack of published information about POLEs and other nonlaboratory research, workshop attendees proposed that greater transparency from the Home Office and SNCOs would be desirable. However, this would still not include activities such as trapping and marking of animals that require no licensing under either the A(SP)A or the WCA (see Figure 1). Understanding the extent of such work would instead require the formation of a new centralized database where such projects could be registered, and data returned and collated. There may be several challenges to creating and maintaining such a database. For example, it is unclear how this would be funded and kept secure, and which organization would be responsible for maintaining it.

### 3.3. Ethical Review

The A(SP)A requires all licensed establishments to have an Animal Welfare and Ethical Review Body (AWERB); the AWERB is generally based in a research institution like a university, company, or government body, and reviews A(SP)A license applications before they are sent to the Home Office for approval, revision, or rejection [1]. Discussion amongst workshop contributors centered around the idea of encouraging AWERBs, and similar ethical review bodies in other countries, to pay greater attention to POLEs and other nonlaboratory research. Some suggested that this might involve drawing the attention of AWERBs to how regulation and research practice work differently in and outside of the laboratory (see [2,5,39] for similar suggestions in the USA). 

This proposal might also involve extending the scope of AWERBs, which are legally mandated committees responsible for overseeing research under the A(SP)A, to cover research not regulated under the A(SP)A such as that conducted overseas and on animal taxa not protected by the A(SP)A (e.g., insects). The AWERBs might also be encouraged to examine subthreshold research, such as the trapping and marking of animals. This may be particularly important given that work of this kind may require no legal oversight or justification, if the animals involved are not protected by the WCA and other wildlife-focused regulations [18]. 

Attendees observed that some AWERBs already extend their mandates in this manner, although the wider scale of this extension is unknown. Workshop contributors proposed that this extension of scope might be encouraged across all AWERBs in the UK and internationally in equivalent bodies. This echoes the proposal that AWERBs should be encouraged to go beyond their “minimum” legally required tasks also to undertake “additional tasks” such as “acting as a forum for discussion and development of ethical advice to the establishment licence holder” [49,50]. It was also observed that peer-reviewed journals are increasingly involved in ethical oversight of research with animals both in and outside of the laboratory, with some major journals releasing their own ethics statements (e.g., *Animal Behaviour* [51]) or requiring ethics statements from authors (e.g., *PLoS ONE* [52]).

### 3.4. Training, Competency, and Support

In other areas of animal research, PHW noted that support organizations or support mechanisms exist. Discussion then centered around the observation that, by contrast, researchers who work at POLEs may lack support networks where they can discuss with their peers practical and ethical issues arising from their animal work. The idea of creating such a support network was discussed, perhaps drawing on the experiences of other bodies such as the LAVA and IAT. 

Researchers at POLEs may also work alongside other actors, such as volunteer citizen scientists in wildlife research who might assist with research activities such as animal trapping not falling under the A(SP)A [2]. Questions focused on training and competency in such circumstances; for example, to what extent should researchers retrain or oversee volunteers? Some citizen scientists may be more highly skilled in trapping and marking animals than the professional researchers working under the A(SP)A with whom they collaborate [53,54]. Even then, ensuring sufficient training and competency of those involved in research is, with good reason, a key requirement of licenses under the A(SP)A [1]. Some challenged the relevance of many A(SP)A training programs for researchers working at POLEs; for example, researchers studying animals not commonly found in laboratories are unlikely to have the opportunity to handle their study species during formal training sessions. However, efforts are being made to create more relevant training programs, such as the Wild Mammal and Wild Bird Home Office Training Course offered by the APHA. 

This, in turn, led to a discussion of variable standards of training in relation to animal research falling outside of the A(SP)A across different animal taxa. For example, ringing training by the BTO is widely considered to be thorough for citizen scientists working with birds. Arguably, such ringing training is more intense than that undertaken by researchers carrying out procedures on animals under the A(SP)A, with ringing training generally taking years rather than weeks and involving considerable experience in the capture and handling of free-living birds in the field. However, training requirements are far more variable for research on nonavian taxa [18]. For example, to catch animals protected under specific schedules of the WCA or (at least while EU laws continue to apply in the UK) the European Habitats Directive (e.g., all bats (Chiroptera), water voles (*Arvicola terrestris*), and great crested newts (*Triturus cristatus*)) for research purposes, applicants usually need to include references and evidence of training or previous experience to support their applications [55]. On the other hand, many species are not protected under relevant schedules of the WCA or other wildlife-focused laws, such as the European rabbit (*Oryctolagus cuniculus*), red fox (*Vulpes vulpes*), and wood mouse (*Apodemus sylvaticus*). The AWA still applies, but no license, training, or demonstrated competency is required to disturb these unprotected animal species, such as via capture and handling (Figure 1). Training is important to help to mitigate the potentially negative consequences of trapping, handling, and other disturbance of wildlife [9]. For example, trapping is widely perceived as the most stressful element of research for a wild animal, with the level of stress animals experience akin to predation [56] and associated mortality potentially high [57]. 

### 3.5. Regulation and Cultures of Care

Discussions also highlighted two specific areas of concern where greater oversight of research might be required, including a consideration of both the purpose of the research and the ethics of conducting it. First, there is minimal regulation of non-A(SP)A trapping and marking for certain wildlife species (e.g., those not protected by the WCA or other wildlife laws, and which can be caught and marked without requiring sedation), meaning that such work may require little training and scientific justification. Following the workshop, three of the presenters (JL, RD, and SJR) engaged in a panel discussion organized by AnNex, which explored the regulation and ethics of wildlife-focused citizen science in greater detail [18]. 

The second area proposed for greater oversight is the work of veterinarians, specifically in taking samples for nontreatment purposes and undertaking experimental or highly novel veterinary treatments. Some workshop contributors argued that there is a need to revise the guidelines issued by the Royal College of Veterinary Surgeons (RCVS) around the distinction between research and RVP. Furthermore, some suggested clarification of the meaning of “immediate group” in these guidelines, a phrase used in the context of indicating that RVP includes only activities that benefit the individual animal patient or its immediate group [58]; however, it is unclear whether “immediate group” refers to species, breeds, herds, or finer scales such as social units.

In relation to both of these areas of concern, questions were raised about whether regulation is the best way to ensure that those involved (e.g., citizen scientists, ecological researchers/consultants, veterinarians) think carefully about the ethics and purpose of their work. The idea was raised in the workshop that the A(SP)A has the advantage of requiring careful consideration of animal welfare, harms, and benefits [41]. However, concerns were also raised about the risk that extending the A(SP)A or introducing further regulations could prevent beneficial and minimally harmful work from taking place, given that considerable investments of time, energy, money, and potentially training by experts may be required to secure licenses under the A(SP)A. These barriers, along with concerns about negative connotations associated with holding an A(SP)A license, already appear to present a real or perceived obstacle for certain groups such as citizen scientists and zoo workers, who may feel unable to secure A(SP)A licenses [59,60].

Discussion therefore also focused on nonregulatory means of encouraging some of the principles of the A(SP)A, such as care for animal welfare and weighing harms and benefits. In essence, the discussion centered on how best to foster a “culture of care”—which is perhaps best described as a “workplace atmosphere” in which care is encouraged for both animals and humans [61]—in non-A(SP)A research. For example, ZB proposed the promotion of an easy guide, which could take the form of an acronym or mnemonic. Recognizing that these issues relate not just to regulation but also to social values and practices, which may vary cross-culturally (e.g., see [62] for a discussion of cultural variation in attitudes towards birds), discussions also considered the possibility of more extensive animal welfare and ethics education in school curricula.

### 3.6. Defining Science

Further discussion in relation to wildlife studies by citizen scientists and ecological consultants, and experimental veterinary treatment, focused on the rigor of scientific methodology, the quality of the data collected, and how data are subsequently made available, a concern being that these areas could be improved. Furthermore, questions of rigor and methodology were linked to the task of defining the remit of the A(SP)A, since it is intended to apply only to activities undertaken for specified “scientific or educational purposes” [1]. Underlying this is arguably a shared assumption that generating useful scientific data is the main justification for undertaking animal research, although other motivations, such as encouraging greater public engagement with nature in the case of citizen science [18,63,64], were also acknowledged.

While the definition of science in the A(SP)A is formed in relation to intended purpose, popular understandings adopt other definitions such as the idea of science as a method. It was suggested that license applications have sometimes been rejected on the basis of being insufficiently rigorous methodologically, meaning that projects fail the harm-benefit analysis [60]. Furthermore, understandings of science as a vehicle for method development may encourage friction between researchers and regulators, if for example academic scientists perceive research activities in fisheries and agricultural management as involving scientific methodologies (e.g., use of blood or scale samples for genetic testing) when such work does not currently fall under the A(SP)A. That said, workshop discussions focused on enduring boundary disputes regarding the A(SP)A’s remit, with acknowledgement that not all parties (e.g., fisheries managers, citizen scientists) may be aware of the regulations and the circumstances under which they may require licenses to work under the A(SP)A. For this reason, there was agreement that there could be instances when such licenses should have been secured (but were not) prior to nonlaboratory work on animals proceeding.

This observation further supports the proposal of developing tools for encouraging careful consideration of regulation, purpose, and ethics in research to be conducted outside of the A(SP)A. It also supports the idea of further developing training and support networks, which ideally could bring in not only researchers working under the A(SP)A but also citizen scientists and others undertaking subthreshold research.

## 4. Conclusions

The workshop succeeded in providing a space where questions, concerns, and experiences could be shared and discussed in confidence. Indeed, the workshop generated robust discussion and thereby highlighted the value of focusing on the unique challenges posed by animal research carried out at POLEs and in other field-based arenas. However, not all questions were answered by consensus and there remains considerable ground for continued debate. In conclusion, therefore, we identify five key areas that we propose require further discussion and attention, which we describe briefly below.

1. *Support and training*. It was agreed that further support and training networks would be helpful in allowing those working at POLEs under the A(SP)A to share experiences and best practice. Such networks would also ideally connect researchers working under the A(SP)A with those undertaking subthreshold research, such as veterinarians, citizen scientists, and ecological consultants.
2. *Ethical review*. Workshop contributors were supportive of the idea that if they do not do so already, AWERBs should be encouraged to learn about how research may be described, peer-reviewed, approved, and carried out differently at POLEs compared with in laboratories. As part of this discussion, it was proposed that AWERBs could be encouraged to oversee animal research not falling under the A(SP)A such as work that is subthreshold, based overseas, or involving animal taxa such as insects and crustaceans not currently classified as protected under the A(SP)A. Some AWERBs already extend their mandates in this manner, although the scale of this extension is unknown. This echoes the idea that AWERBs should be viewed as hubs for ethics and welfare discussion [49,50].
3. *Cultures of care*. It was proposed that methods for encouraging and ensuring harm−benefit analysis and attention to animal welfare within nonregulated work, such as the marking and tagging of wild animals not protected under the WCA, should be explored. This may involve regulation, or nonregulatory means of encouraging cultures of care such as the distribution of a plainly written and widely accessible guide that prompts consideration of ethics and purpose, although implementation and funding of such activities remain unclear.
4. *Setting of boundaries.* Specific regulatory grey areas identified by some workshop participants relate to the boundaries set between research and practice, such as RVP. It was proposed that ambiguity relating to RVP should be clarified by the RCVS.
5. *Statistics and transparency*. Some workshop attendees felt that it would be helpful to have access to further published statistics on the numbers of research projects and animals worked on in nonlaboratory settings, both for research carried out under the A(SP)A and subthreshold research, such as the trapping and marking of wildlife performed by citizen scientists and ecological consultants. This might require not only action by the Home Office and SNCOs, but also the creation of a new database where work requiring no licenses could be registered. Although it remains unclear how such a project would be implemented, funded, and kept secure, there may be scope for enrolling established citizen science platforms such as Zooniverse (https://www.zooniverse.org/).

Overall, we propose that the unique opportunities and challenges of animal research conducted outside of the laboratory should be a subject of greater discussion and attention. We suggest that the list above offers a useful starting point for policy-makers and others involved in regulating and overseeing animal work in the UK, and in some cases beyond.

## Supplementary Materials

The following are available online at https://www.mdpi.com/2076-2615/10/10/1868/s1, Table S1: Organizers’ and invited presenters’ expertise and role or presentation title during the workshop, in alphabetical order by surname.
