# Supplementary material for: Animal Research beyond the Laboratory: Report from a Workshop on Places Other than Licensed Establishments (POLEs) in the UK
Source: Animals (Basel). 2020 Oct 13;10(10):1868. doi: 10.3390/ani10101868 (PMC7602001; doi:10.3390/ani10101868)
Supplement: Supplementary file 1 [file animals-10-01868-s001.pdf]

animals MDPI

Supplementary Materials: Animal Research beyond the Laboratory: Report from a Workshop on Places Other than Licensed Establishments (POLEs) in the UK

Alexandra Palmer [1,*], Beth Greenhough [1], Pru Hobson-West [2], Reuben Message [1], James N. Aegerter [3], Zoe Belshaw [4], Ngaire Dennison [5], Roger Dickey [6], Julie Lane [3], Jamie Lorimer [1], Kate Millar [7], Chris Newman [8], Kirsten Pullen [9], S. James Reynolds [6,10], Dominic J. Wells [11], Matthew J. Witt [12] and Sarah Wolfensohn [13]

1 School of Geography and the Environment, University of Oxford, Oxford OX1 3QY, UK; beth.greenhough@ouce.ox.ac.uk (B.G.); reuben.message@ouce.ox.ac.uk (R.M.); jamie.lorimer@ouce.ox.ac.uk (J.L.)
2 School of Sociology and Social Policy, University of Nottingham, Nottingham NG7 2RD, UK; Pru.Hobson-west@nottingham.ac.uk
3 National Wildlife Management Centre, Animal and Plant Health Agency, Sand Hutton, York YO41 1LZ, UK; James.Aegerter@apha.gov.uk (J.N.A.); Julie.Lane@apha.gov.uk (J.L.)
4 PDSA Nottingham, Dunkirk Road, Nottingham NG7 2PH, UK; zoevet@hotmail.com
5 Biological Services, University of Dundee, Dundee DD1 5EH, UK; n.dennison@dundee.ac.uk
6 Army Ornithological Society (AOS), Aldershot GU11 1PS, UK; roger.dickey52@gmail.com (R.D.); J.Reynolds.2@bham.ac.uk (S.J.R.)
7 Centre for Applied Bioethics, School of Biosciences and School of Veterinary Medicine and Science (SVMS), University of Nottingham, Nottingham LE12 5PF, UK; kate.millar@nottingham.ac.uk
8 Wildlife Conservation Research Unit, The Recanati-Kaplan Centre, Department of Zoology, University of Oxford, Oxford OX13 5QL, UK; chris.newman@zoo.ox.ac.uk
9 Wild Planet Trust, Paignton Zoo, Totnes Road, Paignton TQ4 7EU, UK; kirsten.pullen@wildplanettrust.org.uk
10 School of Biosciences, University of Birmingham, Birmingham B15 2TT, UK
11 Department of Comparative Biomedical Sciences, Royal Veterinary College, London NW1 0TU, UK; dwells@rvc.ac.uk
12 College of Life and Environmental Sciences, University of Exeter, Exeter EX4 4QD, UK; M.J.Witt@exeter.ac.uk
13 School of Veterinary Medicine, University of Surrey, Guildford GU2 7AL, UK; s.wolfensohn@surrey.ac.uk
* Correspondence: alexandra.palmer@ouce.ox.ac.uk

Table S1. Organizers' and invited presenters' expertise and role or presentation title during the workshop, in alphabetical order by surname.

Name	Affiliation	Expertise	Workshop role / Presentation title
James Aegerter	National Wildlife Management Centre (APHA), York, UK	Wildlife research (bats)	*'Why are you doing it like that?!'*
Zoe Belshaw	The People's Dispensary for Sick Animals (PDSA), Nottingham, UK	Veterinary medicine (evidence-based, small animal internal medicine)	*Where recognised veterinary practice meets A(SP)A*
Ngaire Dennison	Biological Services, University of Dundee, Dundee, UK	Regulation of animal research (general)	*Is it science? POLEs – some grey areas and misconceptions*
Roger Dickey	Army Ornithological Society (AOS), Aldershot, UK	Citizen science (birds)	*Licenced procedures and citizen science in field ornithology: Are they POLEs apart? (with S. James Reynolds)*
Beth Greenhough	School of Geography and the Environment, University of Oxford, Oxford, UK	Social scientist with AnNex, PI overseeing Species and Spaces research strands	Organizer; chair of closing reflections session
Pru Hobson-West	School of Sociology and Social Policy, and School of Veterinary Medicine and Science, University of Nottingham, Nottingham, UK	Social scientist with AnNex, PI overseeing Publics and Professions research strands	Organizer; chair of session on farms, zoos, and marine research
Julie Lane	National Wildlife Management Centre (APHA), York, UK	Wildlife research (ethics, regulation)	*Wild places: The ethical, legal and logistical trials and tribulations of a wildlife researcher*
Jamie Lorimer	School of Geography and the Environment, University of Oxford, Oxford, UK	Social science of human-animal relationships and scientific research	*What makes an animal charismatic, and how does this affect science and politics?*
Reuben Message	School of Geography and the Environment, University of Oxford, Oxford, UK	Social scientist with AnNex, leading Species research strand	Organizer; chair of session on veterinary clinical research
Kate Millar	Centre for Applied Bioethics, School of Biosciences and School of Veterinary Medicine and Science (SVMS),	Agricultural and veterinary ethics	*Ethical pivot points related to experimentation involving farm animals at POLEs: Ethical boundaries, legal places and societal expectations*

	University of Nottingham, Nottingham, UK		
Chris Newman	Wildlife Conservation Research Unit (WildCRU), Department of Zoology, University of Oxford, Oxford, UK	Wildlife research (mammals)	*A square peg in a round hole: A(SP)A, wildlife, and working at POLEs*
Alexandra Palmer	School of Geography and the Environment, University of Oxford, Oxford, UK	Social scientist with AnNex, leading Spaces (i.e. POLEs) research strand	Organizer; chair of session on wildlife research
Kirsten Pullen	Wild Planet Trust, Paignton Zoo, Paignton, UK	Zoo research	*Research in zoos and aquariums: The application of A(SP)A to a specific POLE*
S. James Reynolds	School of Biosciences, University of Birmingham, Birmingham, UK & AOS, Aldershot, UK	Wildlife research (birds)	*Licenced procedures and citizen science in field ornithology: Are they POLEs apart? (with Roger Dickey)*
Dominic Wells	Department of Comparative Biomedical Sciences, Royal Veterinary College, London, UK	Veterinary research	*The RVC experience of operating veterinary research at A(SP)A POLEs*
Matthew Witt	College of Life and Environmental Sciences, University of Exeter, Exeter, UK	Wildlife and fisheries research (marine species)	*POLEs and pelagic animals: the challenges of A(SP)A at sea*
Sarah Wolfensohn	School of Veterinary Medicine, University of Surrey, Guilford, UK	Animal welfare (general)	*Animal research at POLEs. A(SP)A 1986 to 2019: Where to now?*
